# Overlapping Patterns of Rapid Evolution in the Nucleic Acid Sensors cGAS and OAS1 Suggest a Common Mechanism of Pathogen Antagonism and Escape

**DOI:** 10.1371/journal.pgen.1005203

**Published:** 2015-05-05

**Authors:** Dustin C. Hancks, Melissa K. Hartley, Celia Hagan, Nathan L. Clark, Nels C. Elde

**Affiliations:** 1 Department of Human Genetics, University of Utah School of Medicine, Salt Lake City, Utah, United States of America; 2 Basic Sciences Division, Fred Hutchinson Cancer Research Center, Seattle, Washington, United States of America; 3 Department of Computational and Systems Biology, University of Pittsburgh, Pittsburgh, Pennsylvania, United States of America; University of Michigan, UNITED STATES

## Abstract

A diverse subset of pattern recognition receptors (PRRs) detects pathogen-associated nucleic acids to initiate crucial innate immune responses in host organisms. Reflecting their importance for host defense, pathogens encode various countermeasures to evade or inhibit these immune effectors. PRRs directly engaged by pathogen inhibitors often evolve under recurrent bouts of positive selection that have been described as molecular ‘arms races.’ Cyclic GMP-AMP synthase (cGAS) was recently identified as a key PRR. Upon binding cytoplasmic double-stranded DNA (dsDNA) from various viruses, cGAS generates the small nucleotide secondary messenger cGAMP to signal activation of innate defenses. Here we report an evolutionary history of cGAS with recurrent positive selection in the primate lineage. Recent studies indicate a high degree of structural similarity between cGAS and 2’-5’-oligoadenylate synthase 1 (OAS1), a PRR that detects double-stranded RNA (dsRNA), despite low sequence identity between the respective genes. We present comprehensive comparative evolutionary analysis of cGAS and OAS1 primate sequences and observe positive selection at nucleic acid binding interfaces and distributed throughout both genes. Our data revealed homologous regions with strong signatures of positive selection, suggesting common mechanisms employed by unknown pathogen encoded inhibitors and similar modes of evasion from antagonism. Our analysis of cGAS diversification also identified alternately spliced forms missing multiple sites under positive selection. Further analysis of selection on the OAS family in primates, which comprises OAS1, OAS2, OAS3 and OASL, suggests a hypothesis where gene duplications and domain fusion events result in paralogs that provide another means of escaping pathogen inhibitors. Together our comparative evolutionary analysis of cGAS and OAS provides new insights into distinct mechanisms by which key molecular sentinels of the innate immune system have adapted to circumvent viral-encoded inhibitors.

## Author Summary

A pathogen’s ability to infect new individuals within and across species is largely driven by its capacity to hijack cellular machinery and overcome the immune system. Pathogens have evolved multiple means to evade and shut down host immunity. Typically, mechanisms of inactivation involve direct interactions between host and pathogen factors. To escape inhibition over the course of generations, host factors frequently evolve in a manner that disrupts interactions at specific interfaces with pathogen factors. Likewise, pathogens adapt to restore such interactions, and these genetic tug-of-wars have been described as “molecular-arms races.” Here we focus on the adaptation of two critical host immune factors, cGAS and OAS that share identity in protein structures despite very limited genetic similarity. Our analysis identifies a variety of ways, including amino acid changes on protein surfaces, by which these host factors appear to escape pathogen-mediated inhibition. Surprisingly, some amino acid substitutions are located at equivalent sites suggesting that cGAS and OAS may have adapted to evade common pathogen encoded inhibitors. These data also identify protein surfaces that are targeted by viruses to inhibit host immunity. Taken together our results indicate the existence of critical, yet-to-be identified viral antagonists of cGAS and OAS.

## Introduction

Pathogens constantly drive the evolution of populations they infect [1,2]. The burden of pathogens on host fitness results in selective pressure on both genes involved in immunity and host factors that are hijacked to promote infection. Therefore, alleles providing some measure of resistance to infection rapidly sweep through host populations. Evidence of past selective pressure can be observed at the molecular level by analyzing amino acid sequences for orthologous genes from a large number of related species [2,3]. Changes in the rate of nonsynonymous amino acid substitutions (*d*_N_) relative to the rate of synonymous changes (*d*_S_)—also referred to as ω—can indicate recurrent positive selection common to host-pathogen interfaces [2]. Other mechanisms of adaptation might be common at these interfaces as well. For example, evasion might proceed through alternate splicing events that result in isoforms missing surfaces recognized by pathogen inhibitors, but to date few studies have considered alternate mechanisms of adaptive evolution at host-pathogen interfaces.

A set of host genes, termed pattern recognition receptors (PRRs), initiate immune responses upon recognition of pathogen macromolecular structures (Reviewed in [4,5]). Because such genes act as a “first line” of defense against pathogens, they have been subject to many genetic conflicts involving pathogen-encoded inhibitors that drive recurrent positive selection [2,6]. PRRs recognize pathogen-associated molecular patterns (PAMPs), which include double-stranded RNA (dsRNA) and double-stranded DNA (dsDNA) produced by pathogens [4,5]. Multiple pathways have been described in mammals to detect microorganism-derived nucleic acids in the cell with most acting in the cytoplasm [4,5]. Two of these pathways involve the 2’-5’-oligoadenylate synthase (OAS) family of proteins [7] and the recently described cyclic GMP-AMP synthase (cGAS) [8] which appears to share a distant evolutionary relationship with OAS based on extensive overlap of protein structures [9–11]. Because PRRs like OAS and cGAS act as crucial sentinels of infection [7,12,13], we set out to compare mechanisms by which they might adapt to pathogen-encoded inhibitors.

OAS proteins are cytoplasmic dsRNA binding proteins that generate the second messenger 2’-5’ oligoadenylate (2-5A_n_) (where n > = 2 and <20) upon RNA binding [7]. 2-5A leads to the dimerization and activation of the latent ribonuclease (RNase L), which degrades host and viral mRNAs [13]. The core OAS unit consists of a nucleotidyltransferase (NTase) within the ClassI-CCase family and OAS1-C terminal domain [7,14,15]. The OAS family has a volatile evolutionary history across animals involving domain coupling and multiple gene duplication events [16,17]. In primates, the OAS family consists of OAS1, OAS2, OAS3, and the catalytically inactive OASL, while rodent genomes contain 12 described OAS genes, eight of which are OAS1 paralogs [17]. OAS1 has one core OAS unit while OAS2 and OAS3 have two and three conserved core OAS units in tandem, respectively [7]. OASL encodes one OAS unit followed by a C-terminal domain consisting of two ubiquitin-like repeats and is enzymatically inactive [18,19]. Inhibition of RNA and DNA virus replication mediated by OAS proteins has been experimentally demonstrated [13,20,21] and a viral-encoded direct inhibitor of OAS1 has been described [22].

cGAS provides complementary surveillance as a cytoplasmic double-stranded DNA binding protein [12] that appears to dimerize upon binding of dsDNA [22,23,24]. DNA binding leads to the generation of the second messenger 2’-3’-cyclic GMP-AMP, also known as G(2’-5’)pA(3’-5’)p or cGAMP, from ATP and GTP by cGAS [11,25–28]. cGAMP activates the STimulator of Interferon Genes (STING) [25,29–31], which in turn activates transcription of Type I Interferon genes through TBK1-IRF3 signals [8,29]. cGAS has been implicated in the control of DNA viruses [12,32,33] and retroviruses [34,35], which is consistent with a strong preference for dsDNA substrates *in vitro* [12]. cGAS has also been linked to the detection of bacterial DNA [36,37] and even the inhibition of RNA viruses [32,38].

**Fig 1:**
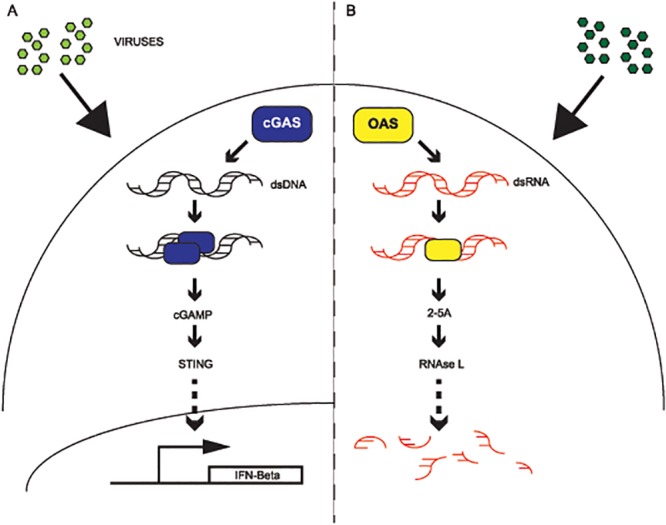
cGAS and OAS1 act in parallel innate defense signaling pathways. **(A)** Model of cGAS signaling. Upon detection and binding of cytoplasmic DNA from viruses (green), cGAS (blue) dimerizes and generates cGAMP, which in turn activates STING signaling (TBK1-IRF3) to promote transcription of interferon beta. **(B)** Model of OAS signaling. Upon detection and binding of double-stranded RNA in the cytoplasm from viruses (green), OAS synthesizes 2–5 oligoadenylate, which activates RNase L and leads to the destruction of viral and cellular RNAs.

The initial characterization of cGAS highlighted several parallels with OAS mediated defenses (Fig 1): 1) nucleic-acid binding, 2) generation of a small nucleotide secondary messenger with a 2’-5’-phosphodiester bond, and 3) viral inhibition. Structural characterization of cGAS revealed that the three-dimensional x-ray crystal structures of OAS1 [14,15] and cGAS share extensive overlap [9–11,39]. In addition, recent structural characterization of the pathogenic protein DncV from *Vibrio cholerae* [40], which also generates cGAMP, but differs in its phosphodiester linkage (A(3’-5’)pG(3’-5’)p) and the reaction order [40,41], suggests a deep evolutionary history of the genes involving extensive sequence and functional divergence.

Here, we focus on more recent evolution of cGAS and OAS to compare how these nucleic acid sensors have been influenced by selection from pathogens. Consistent with their vital role in immune surveillance [8,13,39], we provide comprehensive evidence that cGAS and OAS1 have been under strong, recurrent positive selection in simian primates. We identified rapidly evolving amino acids sites at homologous positions of a common protein surface on cGAS and OAS1 proteins, supporting the surprising possibility of a shared recent evolutionary history of escape from antagonism by common pathogens. In addition, extensive evolutionary analyses of the primate OAS gene family revealed a novel model of adaptation through repeated gene fusion events. Furthermore, we identified multiple alternate spliced forms of cGAS, which maintain intact ORFs, including ones omitting an exon containing rapidly evolving residues. Together these results yield a wealth of insight into mechanisms of adaptive evolution for key nucleic acid sensors acting as a first line of host defenses against diverse pathogens.

## Results

### Rapid evolution of cGAS in primates

Cyclic GMP-AMP synthase (cGAS), previously referred to as C6ORF150, provides a primary block against viruses [12,38] and intracellular bacteria [36,37]. Following binding of cytoplasmic dsDNA, cGAS generates cGAMP [12](Fig 1A), a secondary messenger that activates the interferon response via STING-TBK1-IRF3 signaling [12,25]. Although a study investigating the evolutionary origins of cGAS was recently reported [42] and a limited phylogenetic analysis was conducted [43], little is known about the evolution of cGAS in primates, including humans. Given its crucial role as a DNA sensor triggering innate immunity, and related previous work, we hypothesized that cGAS has been subject to recurrent pathogen-driven evolution in primates.

**Fig 2:**
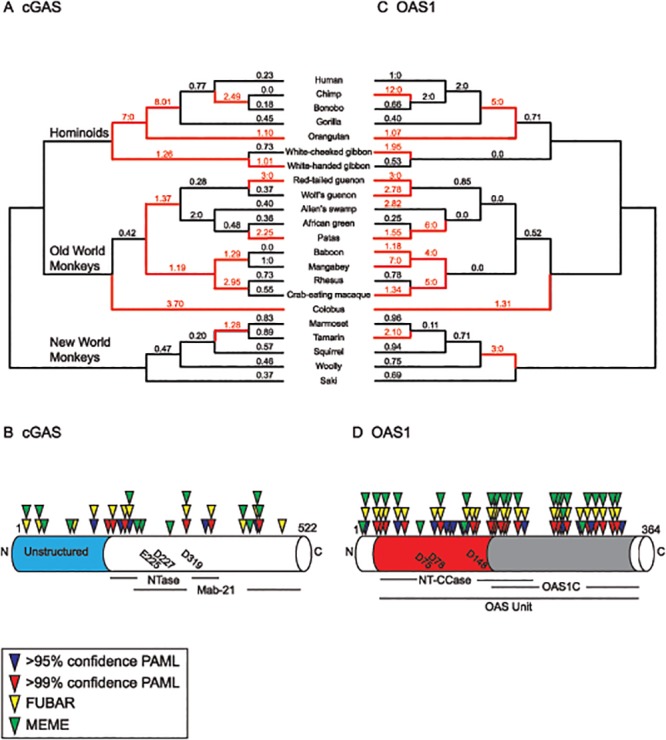
Widespread signatures of positive selection for cGAS and OAS1 across the primate lineage. Phylogenetic analyses of cGAS **(A,B)** and OAS1 **(C,D)** were carried out using sequences from 22 matching primate species. **(A)** A species tree displaying sampled primate sequences for cGAS with dN/dS (ω) values obtained from free-ratio analyses (PAML[45], see Methods) indicated above each branch. ω values > 1 or at least 3 nonsynonymous: 0 synonymous amino acid changes are labeled in red with the corresponding branch (red branch). **(B)** cGAS gene structure with annotated domains and catalytic residues (below). Amino acid sites with statistically significant ω values obtained from NSsites (PAML [44]), FUBAR, and MEME (HyPhy [46]) are indicated above the gene. **(C)** ω values for OAS1 across primate evolution. The species tree is labeled as described for the cGAS tree. **(D)** OAS1 gene structure with amino acids displaying statistical significant ω values. Actual amino acid residue refers to human reference sequence. Catalytic amino acid residues for both cGAS and OAS1 are indicated within the gene diagram.

To determine if cGAS evolved under positive selection in primates, we cloned and sequenced cDNA of cGAS from 22 simian primates (which includes several available primate cGAS sequences from public databases; see Methods and S1 Dataset) to obtain a dataset representing approximately 40 million years of divergence (Fig 2A). Next, we used a combination of maximum likelihood-based algorithms to assess ratios of non-synonymous to synonymous substitution rates (*d*_N_/*d*_S_). The sites model implemented in Phylogenetic Analysis by Maximum Likelihood (PAML) [44] calculates *d*_N_/*d*_S_ values per amino acid position and compares models that omit or accommodate elevated *d*_N_/*d*_S_ to test for positive selection. Our alignment of primate cGAS orthologs revealed signatures of positive selection (p-value <0.0001) (S1 Table and S1 Fig). We further analyzed cGAS variants using the PARtitioning approach for Robust Inference of Selection (PARRIS) algorithm from the HyPhy package [45], which also accounts for recombination events in the dataset, as well as BUSTED, a related measure to detect gene wide evidence of positive selection [46]. PARRIS and BUSTED revealed complementary evidence for positive selection on cGAS in the primate lineage (p<0.017 and p<0.001 respectively) (S2 Table and S3 Table).

To investigate whether cGAS has been subject to episodic positive selection during primate evolution, we calculated *d*_N_/*d*_S_ values at each branch in our primate phylogeny using the free-ratio model in PAML. Consistent with a critical role as a host defense gene antagonized by specific viral inhibitors, cGAS exhibits *d*_N_/*d*_S_ ratios exceeding one—a hallmark of positive selection—on various branches in hominoid, Old World, and New World monkey lineages (Fig 2A). The branch separating ancestors of orangutans from humans, chimps, bonobos, and gorillas in the hominoid lineage was especially remarkable for its inferred episode of positive selection (*d*_N_/*d*_S_ = 8.01, 22 inferred nonsynonymous (N): 1 synonymous (S) amino acid changes). We carried out complementary analysis of episodic selection using the GA-Branch and aBSREL test in HyPhy [47](S2 Fig and S3 Fig), which also supports a history of episodic positive selection on cGAS in primates.

**Fig 3:**
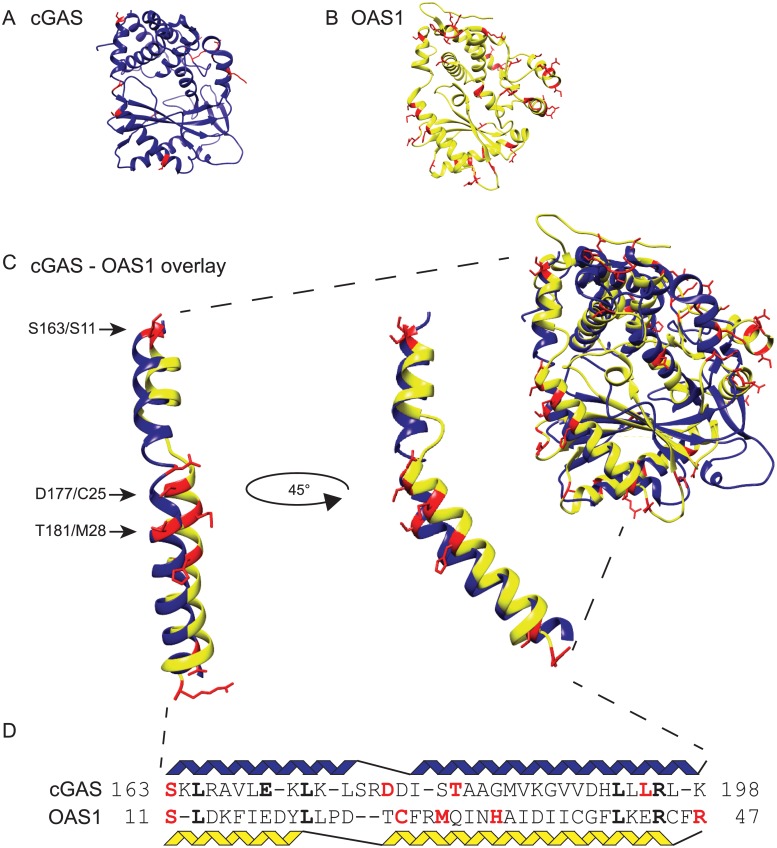
Structurally related cGAS and OAS1 proteins share positions under positive selection. Sites under positive selection (red)(Fig 2B and Fig 2C), were mapped onto the apo crystal structure of human cGAS (blue) **(A)** (PDB: 4KM5)[9] and human OAS1 (yellow) **(B)**(PDB: 4IG8)[14]. **(C)** The cGAS and OAS1 crystal structures were merged using Chimera [69] to visualize structural overlap. The merge of the helical spine region of cGAS and OAS1 reveals overlap of at least three sites under positive selection. Black arrows indicate shared sites with the human reference sequence amino acids for cGAS/OAS1. **(D)** An amino acid sequence alignment of cGAS and OAS1 highlights shared sites under positive selection (red) and sequence identity (bold).

Next we analyzed single amino acid sites in cGAS with evidence of positive selection. Amino acid positions with a *d*_N_/*d*_S_ > 1 in innate immune factors have been experimentally demonstrated in several cases to be sites critical for protein-protein interactions between host and pathogen proteins [2,6]. Multiple amino acid sites in cGAS were inferred to have a *d*_N_/*d*_S_ ratio significantly greater than 1 (Fig 2B). The sites are distributed throughout the protein, a pattern common to other antiviral proteins [2]. Taking advantage of structural studies of cGAS, we mapped sites of selection to a solution of the crystal structure (Fig 3A and S4 Fig). While the nucleic acid binding domains of other nucleic acid sensors appear under purifying selection [6], we identified two sites under positive selection in cGAS that make contact with DNA (S4 Fig). The remaining sites under positive selection are located at surface exposed residues on four distinct regions of the protein (Fig 3A), consistent with previous observations of other nucleic acid sensors that adapt to evade pathogen-encoded inhibitors [2,6].

### Evolutionary analysis of OAS1 suggests shared evolutionary pressures with cGAS

Biochemical and other experimental approaches have identified parallels between the OAS and cGAS pathways: 1) binding of viral nucleic acids, 2) generation of small nucleotide secondary messengers containing 2’-5’ phosphodiester bonds, and 3) use of these secondary messengers to activate an antiviral response [12,39]. In addition, crystallographic analyses of the cGAS protein [9–11,48] revealed extensive structural homology between OAS1 and cGAS despite limited overall sequence identity (~11% amino acid identity). Given these functional relationships, we hypothesized that cGAS and OAS1 might share similar modes of adaptation in response to viral antagonism. To test this idea, we carried out evolutionary analysis of OAS1 using cDNA sequences from the same panel of 22 primate species considered for our analysis of cGAS (Fig 2C).

Using PAML and PARRIS or BUSTED in HyPhy, we found that OAS1 is under positive selection in primates (p<0.001) (S4–S6 Tables and S5 Fig), consistent with previous reports with smaller datasets [49]. Branch specific analysis revealed multiple nodes across the primate phylogeny with elevated *d*_N_/*d*_S_ values, similar to cGAS (Fig 2C). We observed episodic positive selection of OAS1 in each primate lineage, including a notable bout leading to the chimpanzee lineage (12N:0S). Complementary analysis corroborated these findings (S3 Fig and S6 Fig) supporting a history of recurrent adaption of OAS1 in primates.

Similar to cGAS, multiple amino acid positions are under selection in OAS1 (Fig 2D). Phylogenetic analysis revealed roughly three times as many sites with statistically significant *d*_N_/*d*_S_ ratios compared to our analysis of cGAS. The complementary MEME, and FUBAR tests (HyPhy package) identified multiple residues overlapping with PAML analysis under positive selection in OAS1 (Fig 2D and S7 Table). These sites are distributed throughout the 364 amino acid protein, a pattern reminiscent of the antiviral Protein kinase R (PKR) [6], and consistent with adaptation of OAS1 to many viral inhibitors.

### Structural comparisons reveal a surface with shared sites under positive selection in cGAS and OAS1

The arrangement of sites under positive selection can predict locations of binding interactions between host and pathogen proteins [2,6,50]. We mapped positively selected sites onto published x-ray crystal structures of human cGAS (Protein Data Bank: 4KM5)[9](Fig 3A) and human OAS1 (Protein Data Bank: 4IG8)(Fig 3B)[14] solved in the apo-form, lacking nucleic acid activators and nucleoside triphosphate substrates. Consistent with the idea that rapidly evolving sites are involved in protein-protein interactions, sites with significantly elevated *d*_N_/*d*_S_ mapped to protein surfaces of cGAS (Fig 3A) and OAS1 (Fig 3B). For cGAS, the sites under selection localized to four distinct regions of the protein: 1) helix 1 and 2, also referred to as the helical “spine”, 2) between helix 11 and 12, 3) between β-sheet 4 and 5, and 4) the unstructured N-terminus which was not crystallized [9]. For OAS1 most protein surfaces, including the helical “spine”, contain at least one rapidly evolving site.

Because cGAS and OAS1 share extensive structural homology [9–11,48], we examined an overlay of the structures to determine if any homologous amino acids or surfaces are rapidly evolving in both proteins. A merge of the two crystal structures highlighting sites under positive selection revealed analogous amino acid positions especially evident on the extended helical spine of the proteins. 4/11 sites in cGAS are located within the spine while 5/36 sites are located along the OAS1 spine as identified by PAML. Close examination of the structures (Fig 3C) suggests that three of these sites are analogous based upon the amino acid backbones and the directionality of the side chains: 1) Ser163/Ser11, 2) Asp177/Cys25, and 3) Thr181/Met28 (human amino acid cGAS/amino acid OAS1). Alignment of the cGAS and OAS1 amino acid sequences (Fig 3D) corresponding to the helices of the spine indicate that Ser163/Ser11 is an analogous position. Although the sequence alignment implies that Asp177/Cys25 and Thr181/Met28 may not be shared positions, the structure indicates otherwise. Permutation tests simulating co-occurrence of three analogous sites under positive selection in the helical spine suggest that such a pattern of overlap is unlikely to arise by chance (p<0.001) (see Methods and Materials, S1 Dataset). Therefore, comparing the location of sites under selection on the merged crystal structures identified distinct and overlapping surfaces under positive selection between cGAS and OAS1.

Similar to cGAS, some sites under positive selection in OAS1 (Protein Data Bank: 4IG8) [14] contact dsRNA (S7 Fig). There are two clusters of sites that contact the sugar phosphate backbone (S7 Fig). The first cluster consisting of Arg47 and Cys54 resides at the C-terminus of the spine is in an unstructured loop between helix αN3 and β1 sheet. The second cluster of sites consists of Thr203, Thr247, and His248 with the latter two in an unstructured loop between helix αC5 and αC5. Collectively, these sites are the first noted as being under positive selection at nucleic-acid binding surfaces for both cGAS and OAS1.

The overlap of positions under positive selection in cGAS and OAS1 prompted us to ask if these host defense genes might have a history of shared antagonism by pathogens during primate divergence. To investigate this idea, we took advantage of our datasets with 22 matching species to determine if there was a correlation between *d*_N_/*d*_S_ values on matching branches of the primate lineage. This analysis uncovered evidence of a surprising correlation (R = 0.57; S8 Fig) between *d*_N_/*d*_S_ values.

We also tested the correlation of OAS1 and cGAS *d*_N_/*d*_S_ values using the maximum likelihood method of Clark and Aquadro [51]. This method employs HyPhy to model a linear correlation between the branch *d*_N_/*d*_S_ values of each gene and tests its significance by comparison to a null model with no relationship [52]. A likelihood ratio test between these models supported a correlation between OAS1 and cGAS (P = 0.039) with the slope of in correlation model equal to 0.76. Both this likelihood test and the linear regression of *d*_N_/*d*_S_ estimates above support a positive correlation between OAS1 and cGAS. Together these results reveal unexpected parallels in the evolutionary history of OAS and cGAS.

### Reduced number of sites under positive selection in OAS2 and OAS3

**Fig 4:**
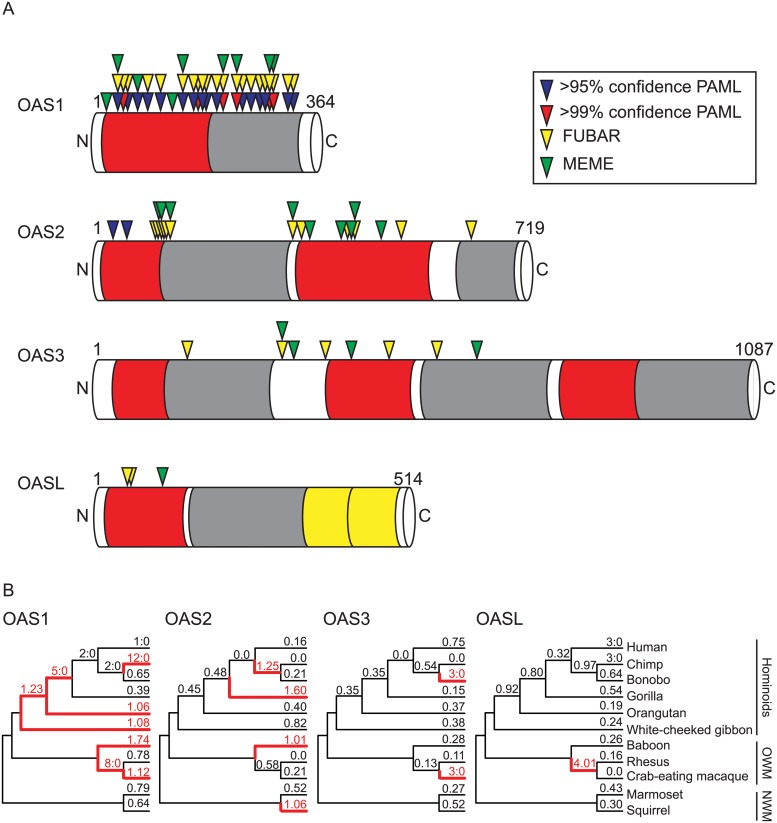
Evolutionary histories vary across the OAS gene family in primates. Phylogenetic analyses of OAS1, OAS2, OAS3, and OASL were carried out using sequences from 11 matching primate species. **(A)** Gene structures of the OAS gene family members in primates. NTase (red) and OAS1-C (gray) domains are indicated. For OASL the ubiquitin-like domains (yellow) are also indicated. Amino acid sites with statistically significant ω values obtained from NSsites (PAML [44]), FUBAR, and MEME (HyPhy [45]) are indicated above the gene. **(B)** Primate species trees with ω values obtained from free-ratio analyses in PAML [44] for each lineage. dN/dS values and lineages with ω > 1 or at least 3 nonsynonymous:0 synonymous amino acid substitutions are highlighted in red.

Given extensive positive selection on OAS1, we set out to gain a more complete view of evolution of OAS genes. OAS1 belongs to a multimember gene family consisting of catalytically active OAS1, OAS2, OAS3 and the catalytically inactive OASL in primates [7]. The OAS genes are distinguished by the number of OAS units, which is the number of NTase and OAS1-C domains they contain through gene fusion events involving genomic tandem duplications (OAS1-1 unit, OAS2-2 units, and OAS3-3 units)(Fig 4A)[7]. Among the OAS family, the enzymatically inactive OASL gene uniquely encodes two ubiquitin repeats at its C-terminus [18,19](Fig 4A). All four members [7] have been implicated in virus inhibition with OAS1, OAS2, and OAS3 directly activating the 2-5A-RNaseL pathway [13] and OASL acting as an enhancer of RIG-I signaling in infected cells [53,54]. Because OAS1 has strong signatures of positive selection on protein surfaces, we were curious whether the other OAS family members also display signatures of positive selection, given the set of genomic fusion events that resulted in proteins that likely bury interacting surfaces.

To determine the evolutionary history of the OAS family in primates, we carried out phylogenetic analysis on a matching panel of primates for all four genes from 11 primates with sequenced genomes and annotated OAS genes (Fig 4B, S9 Fig, and S8–S9 Tables). Consistent with our observations of the more extensive dataset, OAS1 displayed strong evidence of positive selection across these 11 primates (p<0.001). OAS2 also displayed signatures of selection (p<0.014) from analysis by PAML but not from complementary analysis with PARRIS (p = 0.191). A more thorough analysis of OAS2 consisting of 20 species further supports evidence for positive selection by all tests (S10 Table and S11 Table). Moreover, the free-ratio model in PAML identified multiple lineages displaying *d*_N_/*d*_S_ >1 across the 11 primates for both OAS1 and OAS2 (Fig 4B). Notably in the 11 species analysis, 22 OAS1 sites were identified as having statistically significant *d*_N_/*d*_S_ values as compared to only two sites for OAS2 using the PAML sites model (Fig 4A).

In contrast, a comparison of OASL sequences from primates did not exhibit significant signatures of positive selection (p = 0.99), while OAS3 was near the significance cut-off (p = 0.08; S8 Table and S9 Table). A more comprehensive panel of OASL sequences, on par with our analysis of OAS1 and OAS2, also failed to uncover signs of positive selection by all measures tested, including BUSTED (S12 Table). Obtaining a larger panel of OAS3 orthologs was hindered by the large and repetitive nature of the three OAS units encoded by the gene. However, the BUSTED algorithm detected evidence of positive selection in OAS3 (p = 0.024, S13 Table). Analysis of sites under positive selection by PAML, MEME, and FUBAR in matching sets of 11 species for OAS1, OAS2, and OAS3 revealed reduced numbers of sites under selection in inverse correlation with the size of each protein (Fig 4 and S7 Table). Therefore, in the divergence of the OAS family in primates, OAS1 revealed strong signatures of positive selection compared to OAS2 and OAS3, consistent with the hypothesis that gene fusion events might obscure protein surfaces recognized by pathogen-encoded inhibitors.

### Multiple alternately spliced cGAS transcripts

**Fig 5:**
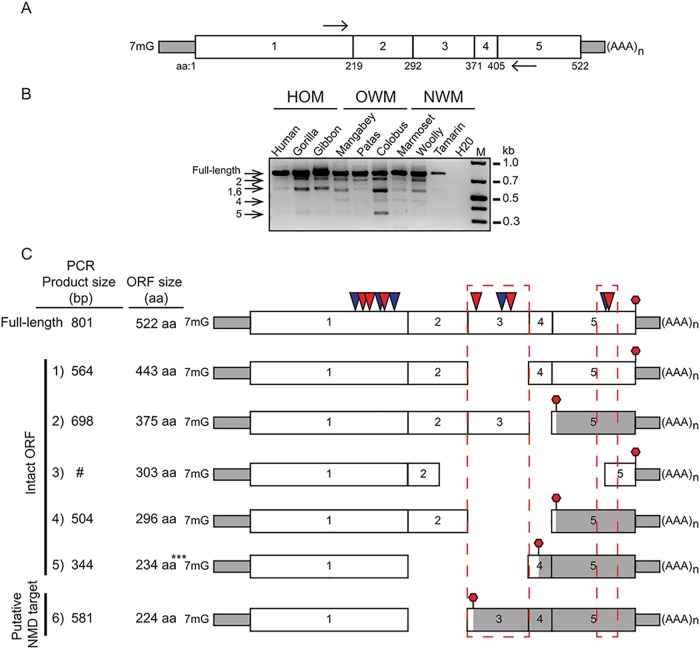
cGAS encodes multiple mRNA spliceforms. **(A)** A schematic of a PCR based assay to detect cGAS spliceforms. A picture of the cGAS cDNA is shown with positions of primers indicated (black arrows). Amino acid positions at each exon-exon junction are labeled below the cGAS gene structure. **(B)** RT-PCR was carried out using cDNA from primate cell lines representing the major primate families: 1) Hominoids (HOM), Old World (OWM), and New World monkeys (NWM). Expected amplicons size representing spliceforms is indicated (arrows left side). Water was used as a negative control. M = 1kb plus DNA standard. **(C)** cGAS spliceforms identified by cDNA and PCR assay. The exon structure of spliceforms is displayed with the location of stop codon (red stop sign). The size of the spliced amplicons in basepairs (bp) and predicted size of the ORFs in amino acids (aa) are labeled next to the corresponding gene structure. #: cDNA was identified by cDNA cloning, but not detected by electrophoresis, ***: stop codon is located 55 nt upstream of last exon-exon junction.

While gene fusions might provide adaptive escape through genetic addition, alternate splicing might provide escape through genetic subtraction. Alternate mRNA spliced variants (spliceforms) are well-documented for contributions to transcript diversity and regulation [55]. Alternative splicing is documented for antiviral proteins, including OAS genes [7]. However, OAS spliceforms have altered C-termini but maintain internal exon structures. By contrast, while cloning cGAS cDNAs, we identified multiple mRNA spliceforms lacking internal exons, some of which encoded intact ORFs. To assess the diversity of cGAS spliceforms across primates, we performed RT-PCR on cDNA extracted from interferon α-treated primary fibroblast cells (Fig 5A and S10 Fig).

We recovered several alternatively spliced cDNAs of cGAS in hominoid, Old World, and New World Monkey species (Fig 5B and S10 Fig), consistent with a varied evolutionary history of transcript variation for cGAS. Sequencing confirmed a diverse set of cGAS mRNA spliceforms (Fig 5C), many of which encode intact open-reading frames. Intriguingly, by comparing spliceform structures to a full-length cGAS gene structure we found cDNAs that lack exon 3, which contains a set of sites under positive selection (Fig 5C). Strikingly, all of the deletions we mapped remove entire helices or beta-strands at linker region boundaries, as opposed to within such domains, consistent with functional roles of the alternately spliced forms (S11 Fig). These cGAS spliceform variants may represent a means to evade or inactivate counteract viral antagonism or perhaps even regulate cGAS.

## Discussion

The Red Queen hypothesis provides a useful framework for investigating recurrent genetic conflicts like those unfolding at host-pathogen interfaces [56]. To date, studying the genetic details of such conflicts has focused on fixed amino acid substitutions in coding regions of genes locked at host-pathogen interfaces. Here we extended such analysis and identified a surprising congruence in cGAS and OAS evolution and also uncovered two potentially adaptive mechanisms involving duplications resulting in gene fusions and alternate splicing of key innate immunity genes.

### Evolution of the OAS family suggests adaptation through gene fusion

OAS proteins are encoded by an ancient and dynamic gene family characterized by extensive duplications in some mammalian lineages [7,16,17]. It is hypothesized that the expansion of the OAS genes involved genomic duplications of the OAS core unit encoded by the first five exons from OAS1 [16]. Because each of these four proteins in primates (OAS1, 2, 3, and L) detect dsRNA from a variety of viruses it is likely that these genes have been involved in genetic conflicts with several inhibitors from different viruses. Consistent with this hypothesis, we identified signatures of positive selection in OAS1 and OAS2, but fewer sites under positive selection in OAS2.

**Fig 6:**
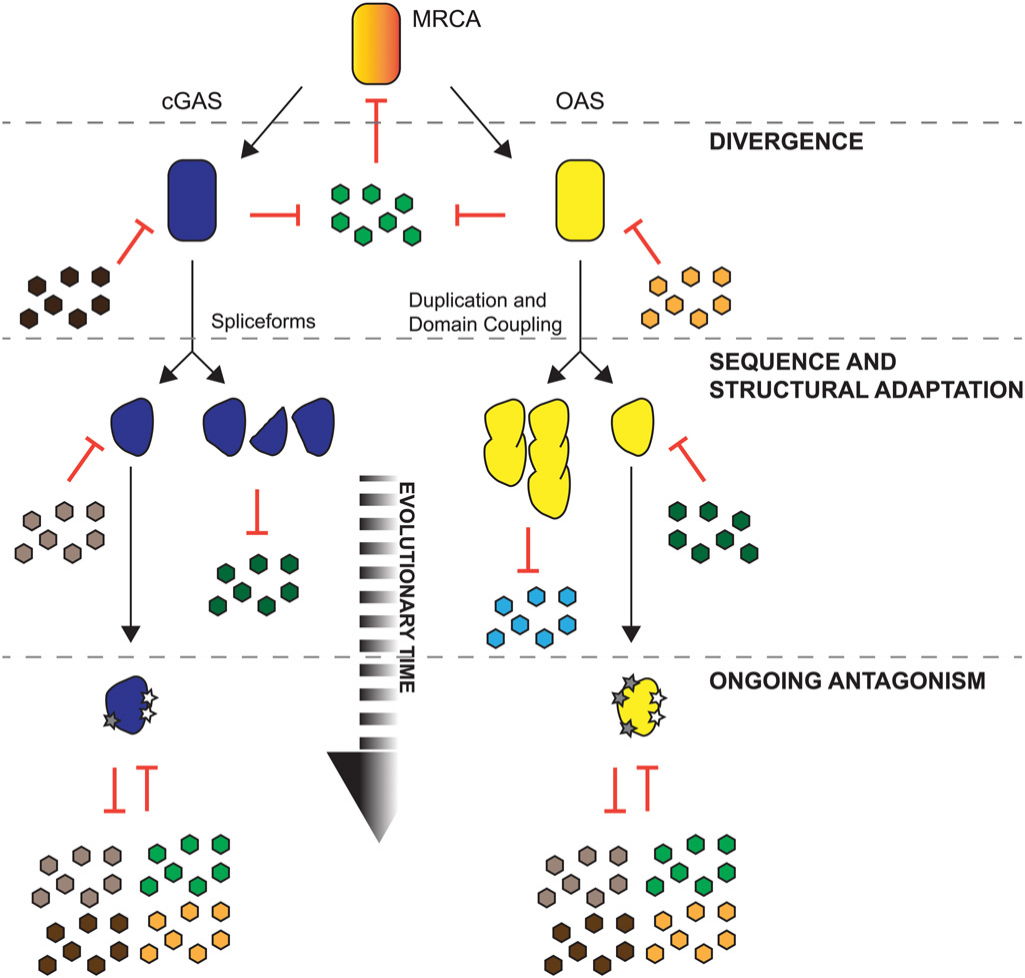
Proposed models for shared and distinct modes of adaptation for cGAS and OAS proteins in primates. An ancestral protein (red) with template independent polymerase activity was challenged by pathogens (green), which led to gene duplications and divergence resulting in ancestral cGAS (blue) and ancestral OAS (yellow). cGAS and OAS likely faced shared and distinct inhibitors encoded by pathogens (colored hexagons). Extensive positive selection of cGAS and OAS resulted in a variety of substitutions that evade inhibition by pathogens. For cGAS, sampling of amino acid substitutions on protein surfaces (gray stars) and the expression of spliceforms that may produce molecular mimics or cGAS variant proteins that evade antagonism could provide diverse mechanisms of escape from pathogen-encoded inhibitors. Some OAS genes also fix amino acid substitutions (gray stars) and may also evade pathogens via duplications and gene fusion events evident in OAS2 and OAS3.

Intriguingly, only a few sites appear under positive selection in OAS3 with even the more sensitive methods of detection (Fig 4 and S7 Table), despite the fact that it synthesizes 2-5A upon dsRNA binding and can robustly block virus replication [7,13,57]. A potential explanation for these observations is that, despite antiviral functions, OAS2 and OAS3 have not been subject to as many pivotal genetic conflicts imposed by pathogen-encoded inhibitors, as is likely for OAS1. Alternately, the domain duplications and gene fusion events that define OAS2 and OAS3 could themselves be adaptive steps in genetic conflicts over the divergence of primates. In this scenario, gene fusions of OAS2 and OAS3 bury protein surfaces via head-to-tail duplications and result in proteins resistant to viral inhibitors that target homotypic interactions (Fig 6). Consistent with this idea is the fact that OAS2 has roughly half as many sites under positive selection as OAS1, and OAS3 half as many as OAS2 (Fig 4 and S7 Table). Furthermore, while OAS1 appears active as a monomer, its activity might be enhanced or modulated by homotypic interactions or self-assembly [58]. As a consequence, some viral inhibitors might act to block OAS1 interactions. Future work will help determine whether, in addition to amino acid substitutions at individual sites under positive selection, gene fusions can provide single mutational steps that obscure protein surfaces from interactions with viral encoded inhibitors.

### Alternative spliced forms of cGAS may evade viral inhibitors

As another potentially adaptive mechanism we identified multiple primate cGAS isoforms that encode intact ORFs. Intriguingly we found four isoforms that cleanly excise all of exon 3 from cGAS, which contains three sites under positive selection. Importantly, spliceforms that lack exon 3 but maintain exon 2 still contain the cGAMP catalytic residues. Based on published cGAS domain deletion data [12] and the presence of catalytic residues, it is possible that all identified cGAS spliceforms retain DNA binding activity owing to the presence of exon 1. In addition, although spliceform 1, 2, and 4 (Fig 5C and S11 Fig) might synthesize cGAMP, it is possible that exon loss may disrupt protein folding. Indeed, it will be necessary to experimentally determine whether any cGAS spliceforms provide adaptive antiviral activity in future work. We posit that these isoforms may serve to remove surfaces antagonized by pathogens, consistent with the loss of several sites under positive selection or that the spliceforms may act as cGAS decoys that bind and sequester viral or bacterial inhibitors.

Regardless of mechanism, alternative splicing has been noted in several cases for evasion of pathogens. Alternative splicing of human APOBEC3G, 3F, and 3H has been documented with varying impacts on antiviral activity and susceptibility to Vif antagonism [59,60]. Supporting the idea that removal of a protein surface may aid in evasion of viral antagonism, one APOBEC3F isoform was noted for resistance to Vif-mediated degradation [59]. On the other hand, another isoform is more susceptible to Vif-mediated degradation [59]. In addition, mutations leading to small deletions have been described for genes targeted by viruses. Of particular interest are a five amino acid deletion in the cytoplasmic tail of human tetherin, which lacks a site under positive selection, that disrupts the functional interaction with the lentivirus encoded antagonist Nef [61], as well as alternately translated forms that resist HIV-1 [62]. Alternatively, it is possible that some of the cGAS spliceforms we identified may serve as antimorphic, negative regulators of cGAS signaling, in a manner analogous to the recently described mini-MAVS variants that modulate the activity of the innate defense factor MAVS [63].

### cGAS and OAS1 have overlapping evolutionary histories in primates

Consistent with their critical role as PRRs [5,64], our analysis indicates that both cGAS and OAS1 are rapidly evolving and reveals a potentially overlapping history of escape from antagonism by common viral inhibitors (Fig 2). Similar to other PRRs known to recognize nucleic acids as substrates [2,6], both cGAS and OAS1 have sites distributed throughout the gene with signatures of positive selection (Fig 2B and Fig 2D). A broad distribution of sites under positive selection is consistent with rapid evolution in response to interactions with inhibitors encoded by multiple pathogens as has been observed for several host defense genes, including the antiviral Protein kinase R [2,6]. That these signatures of adaptive evolution might reflect genetic conflicts with multiple inhibitors is consistent with the fact that OAS1 and cGAS detect multiple pathogens [15,32,33,35,38,65]. Furthermore, although cGAS exhibits only about a third the number of sites under selection compared to OAS1, the robust signatures of selection we observed strongly predict the existence of multiple direct inhibitors of cGAS that have yet to be discovered.

The localization of amino acid positions under positive selection can identify new interfaces involved in protein-protein interactions between host and pathogen factors [2]. Notably, although some protein domains may be dispensable for basal activity in the context of innate immunity, these domains may have as of yet undefined roles in regulation or may be targeted by pathogen factors to inactivate PRRs. For instance, the unstructured N-terminal 160 amino acids of cGAS are dispensable for cGAS activity *in vitro* and *in vivo* [12]. However, we identified several sites under positive selection within the cGAS N-terminus. Although the N-terminus is the least conserved domain of cGAS [12], the statistically significant *d*_N_/*d*_S_ ratios for these sites (posterior probability >0.99) suggest that this domain may be a prime target for pathogen inhibitors of cGAS.

In addition to identifying three structurally homologous rapidly evolving sites along the spine of both OAS1 and cGAS (Fig 3), we find evidence of an intriguing correlation between rates of evolution (*d*_N_/*d*_S_ values) for matching branches in the primate tree (Fig 2A, Fig 2C, and S8 Fig). This correlation of overall rates of evolution suggests that cGAS and OAS1 may have been subject to inhibition on the same primate branches—and perhaps even by the same pathogen or groups of pathogens—over the course of primate divergence. We hypothesize that double-stranded DNA viruses, such as poxviruses that replicate in the cytoplasm, represent strong candidates for encoding such inhibitors because they produce both double-stranded RNA and DNA and deploy inhibitors of immune functions. Consistent with this hypothesis is the observation that some viruses, such as poxviruses, are sensed by both cGAS [12,32,66] and OAS1 [21]. One known herpesvirus inhibitor of OAS1 is Us11 [22], which in light of these data, is also an intriguing candidate that remains to be tested for inhibition of cGAS.

### Conclusions

The recent discovery of cGAS as the basis of a crucial nucleic acid sensing function has generated considerable interest in characterizing this newly described host defense [12,25]. Not only can cGAS sense and respond to a variety of pathogens, it has also been postulated to provide a means of spreading intercellular signals of infection via its generation of the secondary messenger cGAMP [67]. Our evolutionary analysis of cGAS over the divergence of primates is consistent with a vital function for cGAS in countering diverse pathogens. These data further predict the existence of at least several pathogen-encoded inhibitors of cGAS, which will be important to identify and characterize to gain a better understanding of the role of cGAS in countering infections.

Another insight into cGAS evolution was the recent observation of extensive overlap in structure with the nucleic acid sensor, OAS1 [9–11,48]. These data suggest a deep evolutionary connection between the genes and also led us to discover a correlation of positive selection among cGAS and OAS1 during primate evolution as well as shared positions under positive selection. These data suggest a shared history of antagonism by inhibitors deployed by pathogens. Finally, both cGAS and OAS genes appear to adapt by additional mechanisms that drastically alter protein structure through alternate splicing or gene fusion events respectively. Taken together this study reveals central roles for cGAS and OAS genes as key sentinels of host defense in the descent of primates.

## Methods and Materials

### Sequence analysis

DNA Sequences from primates with sequenced genomes were retrieved from the NCBI database using BLAST searches or from the UCSC genome browser (genome.ucsc.edu) using BLAT searches. For other primates, sequences were obtained by Sanger sequencing of PCR amplicons using cDNA as a template or genomic DNA. Briefly, cDNA was synthesized using Superscript III mastermix (Life Technologies) or Maxima cDNA synthesis kit (Thermo) from total RNA extracted from fibroblast cell lines obtained from Coriell. Sequences of interest were PCR amplified from cDNA using Phusion High-Fidelity mastermix (Thermo) according to the manufacturer’s instructions and analyzed by 1–2% agarose gel electrophoresis. Amplicons of interest were excised, purified using Zymo gel extraction kit, and subject to Sanger sequencing or TOPO cloned (Life Technologies) followed by sequencing. For cGAS sequences from New World Monkeys, each exon was PCR amplified from genomic DNA. DNA sequences were analyzed using Geneious software.

DNA sequence alignments were carried out using MUSCLE with default settings in Geneious. All sequences are available in S1 Dataset. Genbank accession numbers KR062003-KR062043.

### Evolutionary analysis

DNA sequences were manually trimmed to remove indels and aligned using Geneious v6.1.7 (Biomatters Ltd.) using default settings. This alignment and a species trees representing currently accepted primate relationships [68] were used as input files for PAML analysis [44] and additional analyses using HyPhy software on Datamonkey.org [45].

We carried out permutation tests by generating two vectors representing cGAS and OAS1 of length 40 to represent 40 amino acids of the helical spine. Executing 1,000,000 trials we determined the probability of getting three sites overlapping between the two vectors (the R script is included in S1 Dataset).

### Modeling

Amino acids identified as being under positive selection using PAML and Datamonkey were mapped onto the three-dimensional crystal structures of the apoform of cGAS (PDB: 4KM5)[9] and DNA co-crystal with mouse cGAS (PDB:406A) [24] and human OAS1 (PDB: 4IG8) [14] using Chimera software (http://www.cgl.ucsf.edu/chimera/)[69].

### RT-PCR

Total RNA from primate fibroblast cell lines treated with 1000 U of interferon/mL was extracted using the RNAeasy kit (Qiagen). 1–2 μg of total RNA was reverse-transcribed using the Maxima cDNA synthesis kit (Thermo). cDNA was diluted to a final volume of 50 μL of which 1 μl was used as a template for PCR. PCR was carried using Phusion according to the manufacturer’s protocol for 35 cycles using cGAS Fint 5’-accgggagctactatgagca-3’ and cGAS Rint 5’-tgtcctgaggcactgaagaa-3’primers. PCR amplicons were analyzed using 2% agarose gel electrophoresis.

## Supporting Information

S1 Fig cGAS gene tree. A phylogenetic tree produced by the PhyML plugin in Geneious using 22 primate cGAS cDNA sequences.
(EPS)

S2 Fig Lineages identified by GA-Branch implemented in HyPhy Datamonkey as rapidly evolving for cGAS. Lineages identified by GA-Branch as being subject to positive selection are labeled in red.
(EPS)

S3 Fig Primate phylograms for A) cGAS and B) OAS1 displaying ω values calculated at branches by aBSREL analysis [47]. In instances where aBSREL was unable to calculate a value (S = 0), the number of nonsynonymous changes relative to synonymous changes calculated by PAML free-ratio analysis are shown. Lineages displaying ω > 1 or at least 3 nonsynonymous changes are highlighted in red.
(EPS)

S4 Fig cGAS protein evolution occurs at both DNA binding pockets. (A) The co-crystal of the mouse cGAS dimer with dsDNA (PDB:406A) with sites under positive selection (red). (B and C) Two interfaces where cGAS sites under positive selection interact with DNA. Each cGAS monomer is individually colored either blue or turquoise. (D) Amino acid alignment of primate variation for the two cGAS rapidly evolving sites that contact DNA in the mouse dimer crystal structure.
(EPS)

S5 Fig OAS1 gene tree. A phylogenetic tree produced by the PhyML plugin in Geneious using 22 primate cGAS cDNA sequences.
(EPS)

S6 Fig Lineages identified by GA-Branch implemented in HyPhy Datamonkey as rapidly evolving for OAS1. Lineages identified by GA-Branch as being subject to positive selection are labeled in red.
(EPS)

S7 Fig Some sites under positive selection in OAS1 are found at the double-stranded RNA binding interface. (A) The co-crystal of OAS1 (yellow) and dsRNA (silver) with sites under positive selection labeled in red. (B and C) Two OAS1 protein surfaces that interact with dsRNA are highlighted. (D) Amino acid alignment showing primate variation for OAS1 at rapidly evolving sites that contact RNA in the crystal structure.
(EPS)

S8 Fig Rates of evolution (measured as the *d*_N_/*d*_S_ ratio) are correlated between the pattern recognition receptors cGAS and OAS1. Models of sequence evolution were used to estimate the rate of each protein on each branch of our primate tree. Plotting each branch by its rate in OAS1 and cGAS reveals a clear linear correlation, indicating that rapid evolution in one gene is typically paralleled by rapid evolution in the other gene. Rates were estimated in the free-ratio branch model of PAML. The correlation analysis was restricted to branches with sufficient divergence to provide reliable estimates of *d*_N_/*d*_S_, specifically those with *d*_S_ > 0.01.
(EPS)

S9 Fig Primate phylogram for OAS gene family displaying ω values calculated at branches by aBSREL analysis [47] . In instances where aBSREL was unable to calculate a value (S = 0), the number of nonsynonymous changes relative to synonymous changes calculated by PAML free-ratio analysis are shown. Lineages displaying ω > 1 or at least 3 nonsynonymous changes are highlighted in red.
(EPS)

S10 Fig RT-PCR of primate cGAS spliceforms using oligo dT primed cDNA template. (A) cGAS RT-PCR splicing assay. (B) cGAS spliceform RT-PCR amplicons resolved by 2% agarose gel electrophoresis. Numbering of spliceforms the same as in Fig 5. Total RNA was isolated from primate fibroblast cell lines (Coriell) using the RNeasy (QIAGEN) kit. First-strand cDNA was synthesized using 4μg of total RNA and Superscript III (Invitrogen) with oligo dT as a primer. cDNA was diluted up to a final volume of 100 μL of which 1μl was used for PCR. PCR amplification was carried out using Phusion (NEB) for 35 cycles. Primer sequences are listed in methods and are the same as those used in Fig 5. α = 24 hour Interferon α treatment, γ = 24 hour Interferon γ treatment, cDNA synthesis was performed using the Maxima cDNA synthesis mastermix (Thermo), using oligo dT for priming, M = 100 bp DNA marker.
(EPS)

S11 Fig cGAS spliceform sequences mapped onto the full-length cGAS structure. cGAS spliceform variant predicted sequences (Fig 5B) are highlighted (B-F) on the crystal structure of human cGAS (PDB:4KM5) [9] (A). Spliceform variant (V) numbering is the same as in Fig 5. Structures in blue indicate remaining sequences following splicing. Silver indicates sequences removed by splicing. Red indicates amino acids identified by PAML analysis as rapidly evolving (see Fig 2). Δ = denotes which exons are removed during mRNA splicing.
(EPS)

S1 Table cGAS gene log likelihood scores and parameter estimates for four models of variable ω among sites assuming the f3x4 model of codon frequencies. (DOCX)

S2 Table Likelihood ratio test statistics for PARRIS analysis of cGAS gene. (DOCX)

S3 Table Likelihood ratio test statistics for BUSTED analysis of cGAS gene (22 species). (DOCX)

S4 Table OAS1 gene log likelihood scores and parameter estimates for four models of variable ω among sites assuming the f3x4 model of codon frequencies. (DOCX)

S5 Table Likelihood ratio test staistics for PARRIS analysis of OAS1 gene. (DOCX)

S6 Table Likelihood ratio test statistics for BUSTED analysis of OAS1 gene (22 species). (DOCX)

S7 Table Rapidly evolving sites identified by evolutionary analysis. (XLSX)

S8 Table OAS gene family evolutionary summary for 11 primate species using PAML. (DOCX)

S9 Table OAS gene family log likelihood scores and parameter estimates for two models of variable ω among sites assuming the f3x4 model of codon frequencies in PAML. (DOCX)

S10 Table OAS2 gene (20 species) log likelihood scores and parameter estimates for four models of variable ω among sites assuming the f3x4 model of codon frequencies. (DOCX)

S11 Table Likelihood ratio test statistics for BUSTED analysis of OAS2 gene (20 species). (DOCX)

S12 Table Likelihood ratio test statistics for BUSTED analysis of OASL gene (21 species). (DOCX)

S13 Table Likelihood ratio test statistics for BUSTED analysis of OAS3 gene (11 species). (DOCX)

S1 Dataset (TXT)

## References

[1] Sawyer SL, Elde NC (2012) A cross-species view on viruses. Curr Opin Virol 2: 561–568. 10.1016/j.coviro.2012.07.003 22835485 PMC3470745

[2] Daugherty MD, Malik HS (2012) Rules of Engagement: Molecular Insights from Host-Virus Arms Races. Annu Rev Genet 46: 677–700. 10.1146/annurev-genet-110711-155522 23145935

[3] Holmes EC (2004) Adaptation and Immunity. PLoS Biol 2: e307 15367941 10.1371/journal.pbio.0020307 PMC516797

[4] Wu J, Chen ZJ (2014) Innate Immune Sensing and Signaling of Cytosolic Nucleic Acids. Annu Rev Immunol 32: 461–488. 10.1146/annurev-immunol-032713-120156 24655297

[5] Orzalli MH, Knipe DM (2014) Cellular sensing of viral DNA and viral evasion mechanisms. Annu Rev Microbiol 68: 477–492. 10.1146/annurev-micro-091313-103409 25002095 PMC4348004

[6] Elde NC, Child SJ, Geballe AP, Malik HS (2009) Protein kinase R reveals an evolutionary model for defeating viral mimicry. Nature 457: 485–489. 10.1038/nature07529 19043403 PMC2629804

[7] Kristiansen H, Gad HH, Eskildsen-Larsen S, Despres P, Hartmann R (2011) The Oligoadenylate Synthetase Family: An Ancient Protein Family with Multiple Antiviral Activities. J Interf Cytok Res 31: 41–47. 10.1089/jir.2010.0107 21142819

[8] Cai X, Chiu Y-H, Chen ZJ (2014) The cGAS-cGAMP-STING pathway of cytosolic DNA sensing and signaling. Mol Cell 54: 289–296. 10.1016/j.molcel.2014.03.040 24766893

[9] Kranzusch PJ, Lee AS-Y, Berger JM, Doudna JA (2013) Structure of Human cGAS Reveals a Conserved Family of Second-Messenger Enzymes in Innate Immunity. Cell Rep 3: 1362–1368. 10.1016/j.celrep.2013.05.008 23707061 PMC3800681

[10] Civril F, Deimling T, de Oliveira Mann CC, Ablasser A, Moldt M, et al (2013) Structural mechanism of cytosolic DNA sensing by cGAS. Nature 498: 332–337. 10.1038/nature12305 23722159 PMC3768140

[11] Gao P, Ascano M, Wu Y, Barchet W, Gaffney BL, et al (2013) Cyclic [G(2'-5')pA(3'-5')p] Is the Metazoan Second Messenger Produced by DNA-Activated Cyclic GMP-AMP Synthase. Cell 153: 1094–1107. 10.1016/j.cell.2013.04.046 23647843 PMC4382009

[12] Sun L, Wu J, Du F, Chen X, Chen ZJ (2013) Cyclic GMP-AMP Synthase Is a Cytosolic DNA Sensor That Activates the Type I Interferon Pathway. Science 339: 786–791. 10.1126/science.1232458 23258413 PMC3863629

[13] Silverman RH (2007) Viral Encounters with 2'-5-'Oligoadenylate Synthetase and RNase L during the Interferon Antiviral Response. J Virol 81: 12720–12729. 10.1128/JVI.01471-07 17804500 PMC2169107

[14] Donovan J, Dufner M, Korennykh A (2013) Structural basis for cytosolic double-stranded RNA surveillance by human oligoadenylate synthetase 1. Proc Natl Acad Sci USA 110: 1652–1657. 10.1073/pnas.1218528110 23319625 PMC3562804

[15] Hartmann R, Justesen J, Sarkar SN, Sen GC, Yee VC (2003) Crystal Structure of the 2′-Specific and Double-Stranded RNA-Activated Interferon-Induced Antiviral Protein 2′-5′-Oligoadenylate Synthetase. Mol Cell Biol 12: 1173–1185. 10.1016/s1097-2765(03)00433-7 14636576

[16] Kumar S, Chandra M, Valente G, Floyd-Smith G (2000) Expansion and Molecular Evolution of the Interferon-Induced 2'-5' Oligoadenylate Synthetase Gene Family. Mol Biol Evol 17: 738–750. 10779534 10.1093/oxfordjournals.molbev.a026352

[17] Perelygin AA, Zharkikh AA, Scherbik SV, Brinton MA (2006) The Mammalian 2′-5′ Oligoadenylate Synthetase Gene Family: Evidence for Concerted Evolution of Paralogous Oas1 Genes in Rodentia and Artiodactyla. J Mol Evol 63: 562–576. 17024523 10.1007/s00239-006-0073-3

[18] Eskildsen S, Justesen J, Schierup MH, Hartmann R (2003) Characterization of the 2'-5-'oligoadenylate synthetase ubiquitin-like family. Nucleic Acids Res 31: 3166–3173. 12799444 10.1093/nar/gkg427 PMC162331

[19] Hartmann R, Olsen HS, Widder S, Jorgensen R, Justesen J (1998) p59OASL, a 2′-5′ oligoadenylate synthetase like protein: a novel human gene related to the 2′-5′ oligoadenylate synthetase family. Nucleic Acids Res 26: 4121–4127. 9722630 10.1093/nar/26.18.4121 PMC147837

[20] Zhao L, Jha BK, Wu A, Elliott R, Ziebuhr J, et al (2012) Antagonism of the Interferon-Induced OAS-RNase L Pathway by Murine Coronavirus ns2 Protein Is Required for Virus Replication and Liver Pathology. Cell Host and Microbe 11: 607–616. 10.1016/j.chom.2012.04.011 22704621 PMC3377938

[21] Rivas C, Gil J, Melkova Z, Esteban M, Diaz-Guerra M (1998) Vaccinia Virus E3L Protein Is an Inhibitor of the Interferon (IFN)-Induced 2-5A Synthetase Enzyme. Virology 243: 406–414. 9568039 10.1006/viro.1998.9072

[22] Sanchez R, Mohr I (2007) Inhibition of Cellular 2'-5' Oligoadenylate Synthetase by the Herpes Simplex Virus Type 1 Us11 Protein. J Virol 81: 3455–3464. 17229694 10.1128/JVI.02520-06 PMC1866071

[23] Li X, Shu C, Yi G, Chaton CT, Shelton CL, et al (2013) Cyclic GMP-AMP Synthase Is Activated by Double-Stranded DNA-Induced Oligomerization. Immunity 39: 1019–1031. 10.1016/j.immuni.2013.10.019 24332030 PMC3886715

[24] Zhang X, Wu J, Du F, Xu H, Sun L, et al (2014) The Cytosolic DNA Sensor cGAS Forms an Oligomeric Complex with DNA and Undergoes Switch-like Conformational Changes in the Activation Loop. Cell Rep 6: 421–430. 10.1016/j.celrep.2014.01.003 24462292 PMC3969844

[25] Wu J, Sun L, Chen X, Du F, Shi H, et al (2013) Cyclic GMP-AMP Is an Endogenous Second Messenger in Innate Immune Signaling by Cytosolic DNA. Science 339: 826–830. 10.1126/science.1229963 23258412 PMC3855410

[26] Diner EJ, Burdette DL, Wilson SC, Monroe KM, Kellenberger CA, et al (2013) The Innate Immune DNA Sensor cGAS Produces a Noncanonical Cyclic Dinucleotide that Activates Human STING. Cell Rep 3: 1355–1361. 10.1016/j.celrep.2013.05.009 23707065 PMC3706192

[27] Ablasser A, Goldeck M, Cavlar T, Deimling T, Witte G, et al (2013) cGAS produces a 2′-5′-linked cyclic dinucleotide second messenger that activates STING. Nature 498: 380–384. 10.1038/nature12306 23722158 PMC4143541

[28] Zhang X, Shi H, Wu J, Zhang X, Sun L, et al (2013) Cyclic GMP-AMP Containing Mixed Phosphodiester Linkages Is An Endogenous High-Affinity Ligand for STING. Mol Cell 51: 226–235. 10.1016/j.molcel.2013.05.022 23747010 PMC3808999

[29] Barber GN (2014) STING-dependent cytosolic DNA sensing pathways. Trends Immunol 35: 88–93. 10.1016/j.it.2013.10.010 24309426

[30] Ishikawa H, Barber GN (2008) STING is an endoplasmic reticulum adaptor that facilitates innate immune signalling. Nature 455: 674–678. 10.1038/nature07317 18724357 PMC2804933

[31] Ishikawa H, Ma Z, Barber GN (2009) STING regulates intracellular DNA-mediated, type I interferon-dependent innate immunity. Nature 461: 788–792. 10.1038/nature08476 19776740 PMC4664154

[32] Schoggins JW, MacDuff DA, Imanaka N, Gainey MD, Shrestha B, et al (2013) Pan-viral specificity of IFN-induced genes reveals new roles for cGAS in innate immunity. Nature: 1–17. 10.1038/nature12862 PMC4077721 24284630

[33] Lam E, Stein S, Falck-Pedersen E (2013) Adenovirus Detection by the cGAS/STING/TBK1 DNA Sensing Cascade. J Virol 88: 974–981. 10.1128/JVI.02702-13 24198409 PMC3911663

[34] Lahaye X, Satoh T, Gentili M, Cerboni S, Conrad C, et al (2013) The Capsids of HIV-1 and HIV-2 Determine Immune Detection of the Viral cDNA by the Innate Sensor cGAS in Dendritic Cells. Immunity: 1–11. 10.1016/j.immuni.2013.07.012 24269171

[35] Gao D, Wu J, Wu Y-T, Du F, Aroh C, et al (2013) Cyclic GMP-AMP synthase is an innate immune sensor of HIV and other retroviruses. Science 341: 903–906. 10.1126/science.1240933 23929945 PMC3860819

[36] Hansen K, Prabakaran T, Laustsen A, Jorgensen SE, Rahbaek SH, et al (2014) Listeria monocytogenes induces IFN expression through an IFI16-, cGAS- and STING-dependent pathway. EMBO J. 10.15252/embj.201488029 PMC4194099 24970844

[37] Zhang Y, Yeruva L, Marinov A, Prantner D, Wyrick PB, et al (2014) The DNA Sensor, Cyclic GMP-AMP Synthase, Is Essential for Induction of IFN- during Chlamydia trachomatis Infection. J Immunol 193: 2394–2404. 10.4049/jimmunol.1302718 25070851 PMC4212656

[38] Schoggins JW, Wilson SJ, Panis M, Murphy MY, Jones CT, et al (2011) A diverse range of gene products are effectors of the type I interferon antiviral response. Nature 472: 481–485. 10.1038/nature09907 21478870 PMC3409588

[39] Hornung V, Hartmann R, Ablasser A, Hopfner K-P (2014) OAS proteins and cGAS: unifying concepts in sensing and responding to cytosolic nucleic acids. Nature Immunol 14: 521–528. 10.1038/nri3719 PMC7097587 25033909

[40] Davies BW, Bogard RW, Young TS, Mekalanos JJ (2012) Coordinated Regulation of Accessory Genetic Elements Produces Cyclic Di-Nucleotides for V. cholerae Virulence. Cell 149: 358–370. 10.1016/j.cell.2012.01.053 22500802 PMC3620040

[41] Kranzusch PJ, Lee ASY, Wilson SC, Solovykh MS, Vance RE, et al (2014) Structure-Guided Reprogramming of Human cGAS Dinucleotide Linkage Specificity. Cell: 1–17. 10.1016/j.cell.2014.03.022 25131990 PMC4157622

[42] Wu X, Wu FH, Wang X, Wang L, Siedow JN, et al (2014) Molecular evolutionary and structural analysis of the cytosolic DNA sensor cGAS and STING. Nucleic Acids Res. 10.1093/nar/gku569 PMC4117786 24981511

[43] George RD, McVicker G, Diederich R, Ng SB, MacKenzie AP, et al (2011) Trans genomic capture and sequencing of primate exomes reveals new targets of positive selection. Genome Res 21: 1686–1694. 10.1101/gr.121327.111 21795384 PMC3202285

[44] Yang Z (2007) PAML 4: phylogenetic analysis by maximum likelihood. Mol Biol Evol 24: 1586–1591. 10.1093/molbev/msm088 17483113

[45] Delport W, Poon AFY, Frost SDW, Kosakovsky Pond SL (2010) Datamonkey 2010: a suite of phylogenetic analysis tools for evolutionary biology. Bioinformatics. 26: 2455–2457. 10.1093/bioinformatics/btq429 20671151 PMC2944195

[46] Murrell B, Weaver S, Smith MD, Wertheim JO, Murrell S, et al (2015) Gene-Wide Identification of Episodic Selection. Mol Biol Evol. 10.1093/molbev/msv035 PMC4408417 25701167

[47] Smith MD, Wertheim JO, Weaver S, Murrell B, Scheffler K, et al (2015) Less Is More: An Adaptive Branch-Site Random Effects Model for Efficient Detection of Episodic Diversifying Selection. Mol Biol Evol. 10.1093/molbev/msv022 PMC4408413 25697341

[48] Kato K, Ishii R, Goto E, Ishitani R, Tokunaga F, et al (2013) Structural and Functional Analyses of DNA-Sensing and Immune Activation by Human cGAS. PLoS ONE 8: e76983 10.1371/journal.pone.0076983 24116191 PMC3792152

[49] Ferguson W, Dvora S, Fikes RW, Stone AC, Boissinot S (2012) Long-term balancing selection at the antiviral gene OAS1 in Central African chimpanzees. Mol Biol Evol. 29: 1093–1103. 10.1093/molbev/msr247 22104212 PMC3341824

[50] Barber MF, Elde NC (2014) Escape from bacterial iron piracy through rapid evolution of transferrin. Science 346: 1362–1366. 10.1126/science.1259329 25504720 PMC4455941

[51] Clark NL, Aquadro CF (2010) A novel method to detect proteins evolving at correlated rates: identifying new functional relationships between coevolving proteins. Mol Biol Evol 27: 1152–1161. 10.1093/molbev/msp324 20044587 PMC2877527

[52] Pond SLK, Frost SDW, Muse SV (2005) HyPhy: hypothesis testing using phylogenies. Bioinformatics. 21: 676–679. 10.1093/bioinformatics/bti079 15509596

[53] Zhu J, Zhang Y, Ghosh A, Cuevas RA, Forero A, et al (2014) Antiviral Activity of Human OASL Protein Is Mediated by Enhancing Signaling of the RIG-I RNA Sensor. Immunity 40: 936–948. 10.1016/j.immuni.2014.05.007 24931123 PMC4101812

[54] Marques J, Anwar J, Eskildsen-Larsen S, Rebouillat D, Paludan SR, et al (2008) The p59 oligoadenylate synthetase-like protein possesses antiviral activity that requires the C-terminal ubiquitin-like domain. J Gen Virol 89: 2767–2772. 10.1099/vir.0.2008/003558-0 18931074

[55] Nilsen TW, Graveley BR (2010) Expansion of the eukaryotic proteome by alternative splicing. Nature 463: 457–463. 10.1038/nature08909 20110989 PMC3443858

[56] vanValen L (1973) A New Evolutionary Law. Evolutionary Theory 1: 1–30.

[57] Ibsen MS, Gad HH, Thavachelvam K, Boesen T, Despres P, et al (2014) The 2'-5' oligoadenylate synthetase 3 (OAS3) enzyme potently synthesizes the 2“-5” oligoadenylates required for RNase L activation. J Virol. 10.1128/JVI.01763-14 PMC4249133 25275129

[58] Ghosh A, Sarkar SN, Guo W, Bandyopadhyay S, Sen GC (1997) Enzymatic Activity of 2“-5-”Oligoadenylate Synthetase Is Impaired by Specific Mutations that Affect Oligomerization of the Protein. J Biol Chem 272: 33220–33226. 9407111 10.1074/jbc.272.52.33220

[59] Lassen KG, Wissing S, Lobritz MA, Santiago M, Greene WC (2010) Identification of Two APOBEC3F Splice Variants Displaying HIV-1 Antiviral Activity and Contrasting Sensitivity to Vif. J Biol Chem 285: 29326–29335. 10.1074/jbc.M110.154054 20624919 PMC2937965

[60] Harari A, Ooms M, Mulder LCF, Simon V (2008) Polymorphisms and Splice Variants Influence the Antiretroviral Activity of Human APOBEC3H. J Virol 83: 295–303. 10.1128/JVI.01665-08 18945781 PMC2612324

[61] Lim ES, Malik HS, Emerman M (2010) Ancient Adaptive Evolution of Tetherin Shaped the Functions of Vpu and Nef in Human Immunodeficiency Virus and Primate Lentiviruses. J Virol 84: 7124–7134. 10.1128/JVI.00468-10 20444900 PMC2898239

[62] Cocka LJ, Bates P (2012) Identification of Alternatively Translated Tetherin Isoforms with Differing Antiviral and Signaling Activities. PLoS Pathog 8: e1002931 10.1371/journal.ppat.1002931 23028328 PMC3460627

[63] Brubaker SW, Gauthier AE, Mills EW, Ingolia NT, Kagan JC (2014) A Bicistronic MAVS Transcript Highlights a Class of Truncated Variants in Antiviral Immunity. Cell 156: 800–811. 10.1016/j.cell.2014.01.021 24529381 PMC3959641

[64] Schneider WM, Chevillotte MD, Rice CM (2014) Interferon-Stimulated Genes: A Complex Web of Host Defenses. Annu Rev Immunol 32: 513–545. 10.1146/annurev-immunol-032713-120231 24555472 PMC4313732

[65] Silverman RH (2007) A scientific journey through the 2-5A/RNase L system. Cytokine Growth F R 18: 381–388. 17681844 10.1016/j.cytogfr.2007.06.012 PMC2075094

[66] Dai P, Wang W, Cao H, Avogadri F, Dai L, et al (2014) Modified Vaccinia Virus Ankara Triggers Type I IFN Production in Murine Conventional Dendritic Cells via a cGAS/STING-Mediated Cytosolic DNA-Sensing Pathway. PLoS Pathog 10: e1003989 10.1371/journal.ppat.1003989 24743339 PMC3990710

[67] Ablasser A, Schmid-Burgk JL, Hemmerling I, Horvath GL, Schmidt T, et al (2013) Cell intrinsic immunity spreads to bystander cells via the intercellular transfer of cGAMP. Nature: 1–17. 10.1038/nature12640 PMC4142317 24077100

[68] Perelman P, Johnson WE, Roos C, Seuánez HN, Horvath JE, et al (2011) A Molecular Phylogeny of Living Primates. PLoS Genet 7: e1001342 10.1371/journal.pgen.1001342 21436896 PMC3060065

[69] Pettersen EF, Goddard TD, Huang CC, Couch GS, Greenblatt DM, et al (2004) UCSF Chimera: A visualization system for exploratory research and analysis. J Comput Chem 25: 1605–1612. 15264254 10.1002/jcc.20084

