# Supplementary material for: Overlapping Patterns of Rapid Evolution in the Nucleic Acid Sensors cGAS and OAS1 Suggest a Common Mechanism of Pathogen Antagonism and Escape
Source: PLoS Genet. 2015 May 5;11(5):e1005203. doi: 10.1371/journal.pgen.1005203 (PMC4420275; doi:10.1371/journal.pgen.1005203)
Supplement: S1 Table — (DOCX) [file pgen.1005203.s012.docx]

| **Table S1:** cGAS gene log likelihood scores and parameter estimates for four models of variable *ω* among sites assuming the f3x4 model of codon frequencies. | | | | | |  |
| --- | --- | --- | --- | --- | --- | --- |
| Site Model | Parameter Estimates | | | | *ℓ* |  |
| M1: neutral | (*ω*_0_= 0) | *f*_0_= | 0.475 |  | -5428.57 |  |
| M2: selection | (*ω*_0_= 0) | *f*_0_= | 0.430 |  | -5403.26 |  |
|  | (*ω*_1_= 1) | *f*_1_= | 0.511 |  |  |  |
|  | ***ω*_2_ = 4.694** | (*f*_2_= 0.0592) |  |  |  |  |
|  | average *d*N/*d*S for each branch = 0.836 | | | |  |  |
| M7: β | p = 0.029 |  | *q*= 0.017 |  | -5429.42 |  |
|  | average dN/dS for each branch = 0.612 | | | |  |  |
| M8: β and *ω*>1 | *p* = | 0.206 | *q* = | 0.147 | -5403.31 |  |
|  | *f*_0_ = | 0.937 |  |  |  |  |
|  | ***ω*_1_ = 4.591** | **(*f*_1_ = 4.591)** |  |  |  |  |
|  | average *d*N/*d*S for each branch = 0.834 | | | |  |  |
