# Supplementary material for: Overlapping Patterns of Rapid Evolution in the Nucleic Acid Sensors cGAS and OAS1 Suggest a Common Mechanism of Pathogen Antagonism and Escape
Source: PLoS Genet. 2015 May 5;11(5):e1005203. doi: 10.1371/journal.pgen.1005203 (PMC4420275; doi:10.1371/journal.pgen.1005203)
Supplement: S2 Table — (DOCX) [file pgen.1005203.s013.docx]

| **Table S2:** Likelihood ratio test statistics for PARRIS analysis of cGAS gene. | | | | | |  |  |  |
| --- | --- | --- | --- | --- | --- | --- | --- | --- |
|  | *ℓ* | 2δ | P-value | Parameter estimates | |  |  |  |
| Null Model (M1): no selection | -5394.81 |  |  |  |  |  |  |  |
| Alternative model (M2): selection | -5390.76 | 8.090 | 0.018 | *ω*_0_= 0.36 | *(f*_0_= 0.686) |  |  |  |
|  |  |  |  | *ω*_1_= 1.00 | *(f*_1_= 0.000) |  |  |  |
|  |  |  |  | ***ω*_2_ = 1.87** | **(*f*_2_= 0.314)** |  |  |  |
|  |  |  |  |  |  |  |  |  |
|  |  |  |  |  |  |  |  |  |
|  |  |  |  |  |  |  |  |  |
|  |  |  |  |  |  |  |  |  |
|  |  |  |  |  |  |  |  |  |
|  |  |  |  |  |  |  |  |  |
|  |  |  |  |  |  |  |  |  |
