# Supplementary material for: Overlapping Patterns of Rapid Evolution in the Nucleic Acid Sensors cGAS and OAS1 Suggest a Common Mechanism of Pathogen Antagonism and Escape
Source: PLoS Genet. 2015 May 5;11(5):e1005203. doi: 10.1371/journal.pgen.1005203 (PMC4420275; doi:10.1371/journal.pgen.1005203)
Supplement: S3 Table — (DOCX) [file pgen.1005203.s014.docx]

| **Table S3:** Likelihood ratio test statistics for BUSTED analysis of cGAS gene (22 species). | | | | | |
| --- | --- | --- | --- | --- | --- |
| Evidence of episodic diversifying selection = Yes | | | | p-value = 0.000 | |
| Model | *log* L | AIC_c_ | ω_1_ | ω_2_ | ω_3_ |
| Unconstrained Model | -5418.77 | 10958.24 | 0.76 (93%) | 0.19 (6.3%) | 69.0 (0.28%) |
| Constrained Model | -5430.34 | 10979.36 | 1.00 (70%) | 0.00 (26%) | 1.00 (4.5%) |
