# Supplementary material for: Overlapping Patterns of Rapid Evolution in the Nucleic Acid Sensors cGAS and OAS1 Suggest a Common Mechanism of Pathogen Antagonism and Escape
Source: PLoS Genet. 2015 May 5;11(5):e1005203. doi: 10.1371/journal.pgen.1005203 (PMC4420275; doi:10.1371/journal.pgen.1005203)
Supplement: S4 Table — (DOCX) [file pgen.1005203.s015.docx]

| **Table S4:** OAS1 gene log likelihood scores and parameter estimates for four models of variable *ω* among sites assuming the f3x4 model of codon frequencies. | | | | | |
| --- | --- | --- | --- | --- | --- |
| Site Model | Parameter Estimates | | | | *ℓ* |
| M1: neutral | (*ω*_0_= 0) | *f*_0_= | 0.581 |  | -3602.26 |
| M2: selection | (*ω*_0_= 0) | *f*_0_= | 0.544 |  | -3535.17 |
|  | (*ω*_1_= 1) | *f*_1_= | 0.284 |  |  |
|  | ***ω*_2_ = 5.420** | (*f*_2_= 0.173) |  |  |  |
|  | average *d*N/*d*S for each branch = 1.220 | | |  |  |
| M7: β | p = 0.005 |  | *q* = 0.00746 |  | -3602.41 |
|  | average dN/dS for each branch = 0.6124 | | |  |  |
| M8: β and *ω*>1 | *p* = | 0.012 | *q* = | 0.025 | -3535.43 |
|  | *f*_0_ = | 0.817 |  |  |  |
|  | ***ω*_1_ = 5.221** | **(*f*_1_ = 0.183 )** |  |  |  |
|  | average *d*N/*d*S for each branch = 1.207 | | |  |  |
