# Supplementary material for: Overlapping Patterns of Rapid Evolution in the Nucleic Acid Sensors cGAS and OAS1 Suggest a Common Mechanism of Pathogen Antagonism and Escape
Source: PLoS Genet. 2015 May 5;11(5):e1005203. doi: 10.1371/journal.pgen.1005203 (PMC4420275; doi:10.1371/journal.pgen.1005203)
Supplement: S6 Table — (DOCX) [file pgen.1005203.s017.docx]

| **Table S6:** Likelihood ratio test statistics for BUSTED analysis of OAS1 gene (22 species). | | | | | |
| --- | --- | --- | --- | --- | --- |
| Evidence of episodic diversifying selection = Yes | | |  | p-value = 0.000 | |
| Model | *log* L | AIC_c_ | ω_1_ | ω_2_ | ω_3_ |
| Unconstrained Model | -3612.28 | 7345.56 | 0.09 (41%) | 0.15 (41%) | 6.02 (18%) |
| Constrained Model | -3624.52 | 7368.01 | 0.00 (6.7%) | 1.00 (4.8%) | 1.00 (88%) |
