# Supplementary material for: Overlapping Patterns of Rapid Evolution in the Nucleic Acid Sensors cGAS and OAS1 Suggest a Common Mechanism of Pathogen Antagonism and Escape
Source: PLoS Genet. 2015 May 5;11(5):e1005203. doi: 10.1371/journal.pgen.1005203 (PMC4420275; doi:10.1371/journal.pgen.1005203)
Supplement: S8 Table — (DOCX) [file pgen.1005203.s019.docx]

| **Table S8:** OAS gene family evolutionary summary for 11 primate species using PAML. | | | |  |
| --- | --- | --- | --- | --- |
| Gene | P-value | Branches^*^ | Sites (>95%)^**^ |  |
| OAS1 | < 0.001 | 8 | 22 |  |
| OAS2 | < 0.014 | 4 | 2 |  |
| OAS3 | < 0.08 | 2 | 0 |  |
| OASL | ~ 0.99 | 1 | 0 |  |
| ^*^The number of branches (red) with *d*N/*d*S > 1 identified by Free Ratio analyses in PAML (see Figure). | | | |  |
|  |  |  |  |  |
| ^**^The number of amino acid sites with 95% or greater statistical signifcance identified by Bayes Emprical Bayes analysis in PAML NSsites (M7 vs. M8). | | | |  |
|  |  |  |  |  |
