# Supplementary material for: Overlapping Patterns of Rapid Evolution in the Nucleic Acid Sensors cGAS and OAS1 Suggest a Common Mechanism of Pathogen Antagonism and Escape
Source: PLoS Genet. 2015 May 5;11(5):e1005203. doi: 10.1371/journal.pgen.1005203 (PMC4420275; doi:10.1371/journal.pgen.1005203)
Supplement: S9 Table — (DOCX) [file pgen.1005203.s020.docx]

| **Table S9:** OAS gene family log likelihood scores and parameter estimates for two models of variable ω among sites assuming the f3x4 model of codon frequencies in PAML. | | | | | | |
| --- | --- | --- | --- | --- | --- | --- |
| Gene | Site Model | Parameter Estimates | | | | *ℓ* |
| OAS1 | M7: β | p = 0.005 |  | *q* = 0.00500 |  | -2520.68 |
|  |  | average *d*N/*d*S for each branch = 0.5000 | | | |  |
|  | M8: β and *ω*>1 | *p* = | 23.569 | *q* = | 99.000 | -2495.54 |
|  |  | *f*_0_ = | 0.786 |  |  |  |
|  |  | ***ω*_1_ = 4.648** | **(*f*_1_ = 0.214 )** |  |  |  |
|  |  | average *d*N/*d*S for each branch = 1.144 | | | |  |
| OAS2 | M7: β | p = | 0.005 | *q* = | 0.005 | -5455.54 |
|  |  | average *d*N/*d*S for each branch = 0.500 | | | |  |
|  | M8: β and *ω*>1 | *p* = | 0.013 | *q* = | 0.022 | -5451.20 |
|  |  | *f*_0_ = | 0.888 |  |  |  |
|  |  | ***ω*_1_ = 2.384** | **(*f*_1_ = 0.112 )** |  |  |  |
|  |  | average *d*N/*d*S for each branch = 0.619 | | | |  |
| OAS3 | M7: β | p = 0.019 |  | *q* = 0.036 |  | -7053.85 |
|  |  | average *d*N/*d*S for each branch = 0.345 | | | |  |
|  | M8: β and *ω*>1 | *p* = | 0.356 | *q* = | 1.817 | -7051.29 |
|  |  | *f*_0_ = | 0.859 |  |  |  |
|  |  | ***ω*_1_ =1.858** | **(*f*_1_ = 0.141)** |  |  |  |
|  |  | average *d*N/*d*S for each branch = 0.400 | | | |  |
| OASL | M7: β | p = 0.005 |  | *q* = 0.009 |  | -3555.833 |
|  |  | average dN/dS for each branch = 0.395 | | | |  |
|  | M8: β and *ω*>1 | *p* = | 0.014 | *q* = | 0.036 | -3555.833 |
|  |  | *f*_0_ = | 0.857 |  |  |  |
|  |  | ***ω*_1_ = 1.000** | **(*f*_1_ = 0.143 )** |  |  |  |
|  |  | average *d*N/*d*S for each branch = 0.394 | | | |  |
