# Supplementary material for: Overlapping Patterns of Rapid Evolution in the Nucleic Acid Sensors cGAS and OAS1 Suggest a Common Mechanism of Pathogen Antagonism and Escape
Source: PLoS Genet. 2015 May 5;11(5):e1005203. doi: 10.1371/journal.pgen.1005203 (PMC4420275; doi:10.1371/journal.pgen.1005203)
Supplement: S10 Table — (DOCX) [file pgen.1005203.s021.docx]

| **Table S10:** OAS2 gene (20 species) log likelihood scores and parameter estimates for four models of variable ω among sites assuming the f3x4 model of codon frequencies. | | | | | | |
| --- | --- | --- | --- | --- | --- | --- |
| Site Model | Parameter Estimates | | | | Sites* with *ω* > 1 | *ℓ* |
| M1: neutral | (*ω*_0_= 0) | *f*_0_= | 0.546 |  |  | -7092.19 |
| M2: selection | (*ω*_0_= 0) | *f*_0_= | 0.555 |  | 107F | -7076.33 |
|  | (*ω*_1_= 1) | *f*_1_= | 0.378 |  |  |  |
|  | ***ω*_2_ = 3.349** | (*f*_2_= 0.066 ) |  |  |  |  |
|  | average *d*N/*d*S for each branch = 0.610 | | |  |  |  |
| M7: β | p = 0.016 |  | *q* = 0.021 |  |  | -7093.02 |
|  | average dN/dS for each branch = 0.419 | | |  |  |  |
| M8: β and *ω*>1 | *p* = | 0.062 | *q* = | 0.097 | 18W, 27Y, 32T, 36E, 49E, 107F, 108C, 109L, 117N, 456P, 488A, 492L, 533R, 583V, 664L | -7076.16 |
|  | *f*_0_ = | 0.924 |  |  |  |  |
|  | ***ω*_1_ = 3.155** | **(*f*_1_ = 0.076)** |  |  |  |  |
|  | average *d*N/*d*S for each branch = 0.601 | | |  |  |  |
| *Amino acid positions relative to the human OAS2 reference sequence. Sites were obtained from Bayes Empirical Bayes analyses in the NSsites test in PAML (p > 95%). | | | | | | |
