# Supplementary material for: Overlapping Patterns of Rapid Evolution in the Nucleic Acid Sensors cGAS and OAS1 Suggest a Common Mechanism of Pathogen Antagonism and Escape
Source: PLoS Genet. 2015 May 5;11(5):e1005203. doi: 10.1371/journal.pgen.1005203 (PMC4420275; doi:10.1371/journal.pgen.1005203)
Supplement: S11 Table — (DOCX) [file pgen.1005203.s022.docx]

| **Table S11:** Likelihood ratio test statistics for BUSTED analysis of OAS2 gene (20 species). | | | | | |
| --- | --- | --- | --- | --- | --- |
| Evidence of episodic diversifying selection = Yes | | | | p-value = 0.001 | |
| Model | *log* L | AIC_c_ | ω_1_ | ω_2_ | ω_3_ |
| Unconstrained Model | -6737.46 | 13587.41 | 0.51 (75%) | 0.39 (24%) | 18.7 (0.89%) |
| Constrained Model | -6744.14 | 13598.75 | 0.00 (44%) | 1.00 (2.9%) | 1.00 (53%) |
