# Supplementary material for: Overlapping Patterns of Rapid Evolution in the Nucleic Acid Sensors cGAS and OAS1 Suggest a Common Mechanism of Pathogen Antagonism and Escape
Source: PLoS Genet. 2015 May 5;11(5):e1005203. doi: 10.1371/journal.pgen.1005203 (PMC4420275; doi:10.1371/journal.pgen.1005203)
Supplement: S12 Table — (DOCX) [file pgen.1005203.s023.docx]

| **Table S12:** Likelihood ratio test statistics for BUSTED analysis of OASL gene (21 species). | | | | | |
| --- | --- | --- | --- | --- | --- |
| Evidence of episodic diversifying selection = No | | | | p-value = 0.724 | |
| Model | *log* L | AIC_c_ | ω_1_ | ω_2_ | ω_3_ |
| Unconstrained Model | -4636.55 | 9389.74 | 0.11 (67%) | 0.30 (21%) | 2.53 (13%) |
| Constrained Model | -4636.87 | 9388.37 | 0.00 (56%) | 1.00 (2.8%) | 1.00 (41%) |
