# Supplementary material for: Overlapping Patterns of Rapid Evolution in the Nucleic Acid Sensors cGAS and OAS1 Suggest a Common Mechanism of Pathogen Antagonism and Escape
Source: PLoS Genet. 2015 May 5;11(5):e1005203. doi: 10.1371/journal.pgen.1005203 (PMC4420275; doi:10.1371/journal.pgen.1005203)
Supplement: S13 Table — (DOCX) [file pgen.1005203.s024.docx]

| **Table S13:** Likelihood ratio test statistics for BUSTED analysis of OAS3 gene (11 species). | | | | | |
| --- | --- | --- | --- | --- | --- |
| Evidence of episodic diversifying selection = Yes | | | | p-value 0.024 | |
| Model | *log* L | AIC_c_ | ω_1_ | ω_2_ | ω_3_ |
| Unconstrained Model | -7047.74 | 14171.74 | 0.06 (69%) | 0.29 (20%) | 3.30 (11%) |
| Constrained Model | -7051.48 | 14177.21 | 0.00 (60%) | 1.00 (2.2%) | 1.00 (38%) |
